# A novel approach for engineering DHCM/GelMA microgels: application in hepatocellular carcinoma cell encapsulation and chemoresistance research

**DOI:** 10.3389/fbioe.2025.1564543

**Published:** 2025-03-14

**Authors:** Dandan Zhou, Xiaoxiao Li, Wencun Liu, Mingjun Zhang, Ying Cheng, Zhousong Xu, Jian Gao, Yiyang Wang

**Affiliations:** ^1^ Department of Gastroenterology, The Second Affiliated Hospital of Chongqing Medical University, Chongqing, China; ^2^ Department of Geriatric Medicine, Jiulongpo People’s Hospital of Chongqing, Chongqing, China; ^3^ Department of Orthopedics, The Third Affiliated Hospital of Chongqing Medical University, Chongqing, China; ^4^ Tissue Repairing and Biotechnology Research Center, The Third Affiliated Hospital of Chongqing Medical University, Chongqing, China; ^5^ Department of Radiology, Jiulongpo People’s Hospital of Chongqing, Chongqing, China; ^6^ Department of Clinical Laboratory, Jiulongpo People’s Hospital of Chongqing, Chongqing, China

**Keywords:** hepatocellular carcinoma, decellularized extracellular matrix (dECM), photocrosslinkable hydrogel, microgel, chemoresistance

## Abstract

Liver cancer, a highly aggressive malignancy, continues to present significant challenges in therapeutic management due to its pronounced chemoresistance. This resistance, which undermines the efficacy of conventional chemotherapy and targeted therapies, is driven by multifaceted mechanisms, with increasing emphasis placed on the protective role of the tumor microenvironment (TME). The hepatocellular carcinoma extracellular matrix (ECM), a primary non-cellular component of the TME, has emerged as a critical regulator in cancer progression and drug resistance, particularly in hepatocellular carcinoma cell (HCC). In this study, a hybrid biomimetic hydrogel was engineered by integrating decellularized hepatocellular carcinoma matrix (DHCM) with gelatin methacrylate (GelMA) precursors. This composite DHCM/GelMA hydrogel was designed to replicate the physicochemical and functional properties of the hepatocellular carcinoma ECM, thereby offering a biomimetic platform to explore the interactions between HCCs and their microenvironment. Leveraging a custom-designed microfluidic 3D printing platform, we achieved high-throughput fabrication of HCC-encapsulated DHCM/GelMA microgels, characterized by enhanced uniformity, biocompatibility, and scalability. These microgels facilitated the construction of hepatocellular carcinoma microtissues, which were subsequently employed for chemoresistance studies. Our findings revealed that DHCM/GelMA microgels closely mimic the hepatocellular carcinoma tumor microenvironment, effectively recapitulating key features of ECM-mediated drug resistance. Mechanistic studies further demonstrated that DHCM significantly upregulates the expression of Aquaporin 3 (AQP3) in the encapsulated HCCs. This upregulation potentially activates mTOR signaling-associated autophagy pathways, thereby enhancing chemoresistance in HCCs. These biomimetic models provide a robust and versatile platform for studying the underlying mechanisms of drug resistance and evaluating therapeutic interventions. This innovative approach highlights the potential of DHCM/GelMA microgels as a transformative tool in cancer-associated tissue engineering and anticancer drug screening. By enabling detailed investigations into the role of ECM in chemoresistance, this study contributes to advancing therapeutic research and offers promising strategies to overcome drug resistance, ultimately improving clinical outcomes in liver cancer treatment.

## 1 Introduction

Liver cancer, a highly aggressive malignancy, is characterized by significant therapeutic resistance, which severely limits the efficacy of both conventional chemotherapy and targeted therapies. These challenges frequently result in suboptimal treatment outcomes and poor patient prognoses (Villanueva, 2019; Llovet et al., 2021). Drug resistance in liver cancer arises from complex mechanisms, including the overexpression of drug efflux pumps, suppression of apoptosis, and the protective effects of the tumor microenvironment (TME) (Chen et al., 2023). Addressing these multifaceted challenges necessitates an in-depth understanding of resistance mechanisms and the development of innovative, personalized therapeutic strategies.

The extracellular matrix (ECM), the primary non-cellular component of the TME, plays a pivotal role in cancer progression and drug resistance. Recent advances in bioengineering have highlighted the importance of accurately replicating the tumor ECM to facilitate mechanistic studies and therapeutic testing (Hynes, 2009; Mouw et al., 2014). Traditional two-dimensional (2D) cell culture models fail to mimic the intricate architecture and dynamic interactions of the ECM, spurring the adoption of three-dimensional (3D) cell culture systems as superior alternatives (Edmondson et al., 2014). Among these, hepatocellular carcinoma organoids constructed by integrating 3D scaffold materials with hepatocellular carcinoma cells (HCCs) provide a biomimetic microenvironment that faithfully reproduces the conditions of *in vivo* tumors. These organoids preserve tumor heterogeneity and self-renewal capacities, enabling accurate modeling of tumor-ECM interactions and serving as robust platforms for studying drug resistance and performing high-throughput drug screening (Fatehullah et al., 2016; Weeber et al., 2017).

The 3D culture models of hepatocellular carcinoma, such as organoids or microtissues, are highly valuable for *in vitro* research as they more accurately mimic the *in vivo* tumor microenvironment (Breslin and O'Driscoll, 2013; Blidisel et al., 2021). These models provide reliable platforms for studying drug resistance, drug screening, and mechanistic investigations in liver cancer (Langhans, 2018). However, the biomaterials currently used to construct hepatocellular carcinoma organoids or microtissues often lack the compositional and structural properties necessary to replicate the native ECM (Li et al., 2022). This discrepancy leads to significant heterogeneity between the cultured models and actual hepatocellular carcinoma tissues, limiting the reliability of research outcomes and their potential for clinical translation. The native hepatocellular carcinoma ECM not only provides physical support for cells but also regulates key cellular behaviors such as proliferation, migration, differentiation, and drug resistance. These effects are mediated through the ECM’s unique biochemical composition, which includes collagen, laminin, and fibronectin, and its topological structure, such as fiber alignment, porosity, and stiffness (Lu et al., 2012; Pickup et al., 2014). For example, specific ECM components, such as collagen I and IV, promote tumor cell survival and enhance drug resistance by activating integrin-mediated signaling pathways, including PI3K/Akt and MAPK (Levental et al., 2009; Rubashkin et al., 2014). Biomaterials that fail to mimic these ECM characteristics cannot accurately replicate the complexity of the liver cancer microenvironment, leading to discrepancies in gene expression, metabolic profiles, and drug responses between *in vitro* models and *in vivo* tumors.

In recent years, decellularized ECM (dECM)-based biomaterials have emerged as an ideal choice for constructing hepatocellular carcinoma organoids or microtissues due to their ability to retain the biochemical composition and structural properties of native ECM (Cox and Erler, 2011; Soto-Gutierrez et al., 2011). The dECM not only provides biochemical signals that closely resemble those *in vivo* but also facilitates cell-matrix interactions through its biomimetic topological structure, which better simulates the physical and biochemical characteristics of the tumor microenvironment (Lu et al., 2012; Pickup et al., 2014). Studies have demonstrated that hepatocellular carcinoma organoid models based on dECM exhibit improved predictive accuracy in drug sensitivity testing, offering a more reliable platform for anticancer drug screening and mechanistic research (Xie et al., 2022; Airola et al., 2024). Therefore, biomaterials with hepatocellular carcinoma ECM-mimetic properties play a crucial role in the construction of hepatocellular carcinoma organoids and microtissues. By accurately mimicking the composition and structural features of native ECM, these materials significantly enhance the biomimicry of *in vitro* models and the reliability of research outcomes, providing strong support for both fundamental research and clinical translation in liver cancer.

Current research focuses on developing novel biomaterial scaffolds tailored for hepatocellular carcinoma organoids, combining them with innovative fabrication techniques to enhance their application in studying drug resistance and advancing therapeutic development (Fong et al., 2018). Emerging materials such as alginate, gelatin methacrylate (GelMA), and methylcellulose are valued for their biocompatibility and tunable mechanical properties (Shu et al., 2024). However, these materials lack specificity in recapitulating the ECM composition unique to hepatocellular carcinoma tumor, which is enriched in factors that influence drug resistance. Integrating ECM components derived from hepatocellular carcinoma tissues into scaffold materials improves biomimicry, creating organoids that more accurately reflect tumor biology (Yamada and Cukierman, 2007).

Despite the advancements, significant challenges remain in scaling the production and clinical application of hepatocellular carcinoma organoids. Ideal biomaterial scaffolds must exhibit biocompatibility, biodegradability, and mechanical properties that closely replicate the stiffness, morphology, and chemical characteristics of the tumor ECM, facilitating tumor cell adhesion, proliferation, and differentiation (Cox and Erler, 2011). Recent developments in advanced hydrogel fabrication techniques, such as droplet microfluidics and photo-crosslinking, offer promising avenues for constructing liver cancer organoids. Droplet microfluidics provides precise control over hydrogel microsphere properties, while photo-crosslinking allows rapid and accurate scaffold preparation, supporting high-throughput organoid fabrication (Cardoso et al., 2023; Kim et al., 2024).

In the present study, a hybrid biomimetic hydrogel was developed by combining decellularized hepatocellular carcinoma matrix (DHCM) with GelMA precursors. This scaffold achieves dual biomimicry by replicating both the physicochemical and functional properties of the hepatocellular carcinoma ECM. Inspired by the stable microdroplets observed on lotus leaves, we employed droplet microfluidics based on Plateau-Rayleigh instability to fabricate cell-laden microgels. This approach integrates droplet microfluidics, 3D printing, and superhydrophobic surface techniques, enabling the efficient, cost-effective, and scalable production of cell-laden microgels. By dispensing photocrosslinkable hydrogel precursor droplets onto this interface in a controlled arrangement, hydrophobic interactions were used to form uniformly spherical droplets, which were then crosslinked to create stable cell-encapsulated hydrogel systems. Utilizing the novel custom-designed microfluidic 3D-printing platform, high-efficiency fabrication of cell-encapsulated microgels was achieved, enabling the construction of hepatocellular carcinoma microtissues and organoids. These models were successfully applied to investigate drug resistance mechanisms, offering a versatile platform for therapeutic research. As biomaterial scaffold technologies and microgel fabrication techniques continue to evolve, the construction of hepatocellular carcinoma microtissues or organoids is expected to become increasingly sophisticated and clinically relevant. These advancements hold significant potential for transforming anticancer drug screening and personalized treatment, providing new hope for improving therapeutic outcomes and combating drug resistance.

## 2 Materials and methods

### 2.1 Reagents and animals

Tris-HCl, Triton X-100, Span 80, gelatin, methacrylic anhydride, lithium phenyl-2,4,6-trimethylbenzoylphosphinate (LAP), and aprotinin were obtained from Aladdin Biochemical Technology Co., Ltd. (Shanghai, China). 5-Fluorouracil (5FU) was purchased from MedChemExpress (MCE) Co., Ltd. (Shanghai, China). RNase and DNase were obtained from Solarbio Biochemical Technology Co., Ltd. (Shanghai, China). DMEM medium, fetal bovine serum (FBS), penicillin/streptomycin, and trypsin were purchased from Gibco Technology Co., Ltd. (Grand Island Biological Company, United States). DNA quantification assays, Live/Dead staining, and Cell Counting Kit-8 (CCK-8) were obtained from Invitrogen Technology Co., Ltd. (Waltham, United States). Hematoxylin-eosin (HE) and immunofluorescence (IF) staining kits, as well as LC3 antibodies, were sourced from Servicebio Technology Co., Ltd. (Wuhan, China). Nude mice were obtained from the Animal Center of Chongqing Medical University, and all animal procedures were approved by the Institutional Animal Care and Use Committee (IACUC) of Chongqing Medical University (No. IACUC-CQMU-2023-0148).

### 2.2 Synthesis process of DHCM/GelMA hydrogel

The Huh7 cell line, a human-derived HCC line originally established from a well-differentiated hepatocellular carcinoma in a Japanese patient, was procured from Cellverse Co., Ltd. (Shanghai, China). The Huh7 cell line is widely used in research focused on liver cancer biology, drug screening, and molecular studies due to its reproducible growth characteristics and ability to maintain liver-specific functions *in vitro* (Yu et al., 2020). Hence, the Huh7 HCCs were injected subcutaneously in nude mice for axillary tumorigenesis experiments, and tumor tissues were collected 3 weeks later for DHCM preparation. The hepatocellular carcinoma tumors were then dissected into fragments and immersed in a detergent solution containing 10 mM Tris-HCl buffer, 3% Triton X-100, 0.1% EDTA, and 10 KIU/mL aprotinin for 48 h at room temperature. Subsequently, the samples were treated with a nuclease solution of 10 mM Tris-HCl buffer, 0.2 mg/mL RNase, and 0.2 mg/mL DNase for 48 h at 37°C to remove nucleic acids.

To evaluate cell elimination, fresh and decellularized tumor specimens were embedded in paraffin, sectioned into 5 mm slices, and stained with the HE following the manufacturer’s instructions. The tissue specimens were weighed to record the wet weight and digested overnight at 60°C in a phosphate-buffered EDTA solution. The DNA content was then quantified utilizing a total DNA quantification assay following the manufacturer’s instructions. In addition, to assess major ECM components, GAG expression in fresh and decellularized tumor specimens were analyzed using a GAG ELISA kits according to the manufacturer’s instructions. The DHCM was finally lyophilized and ground into powders.

The GelMA hydrogel was prepared through a multi-step process: a 10% (w/v) solution of gelatin was dissolved in phosphate-buffered saline (PBS) at 60°C with continuous stirring. Methacrylic anhydride was then added dropwise over 1 hour, and the reaction proceeded for 4 hours at 60°C before being stopped with concentrated PBS. The mixture was purified through dialysis in a sealed bag at 60°C for a week to remove unreacted methacrylic anhydride. The purified product was frozen at −80°C for 24 h and subsequently lyophilized for 72 h, resulting in a GelMA suitable for applications requiring photopolymerizable biomaterials. The 5% w/v GelMA hydrogel precursors, 5% w/v DHCM powders and 0.25% w/v LAP were mixed and dissolved in PBS at 37°C according to a related previous study (Hua et al., 2022). After crosslinking under ultraviolet (UV) irradiation (365 nm, 850 mW) for 30 s, the DHCM/GelMA hydrogel was obtained.

### 2.3 Fabrication of the cell-laden microparticle

Hydrogel microparticles were synthesized using microfluidic technology, wherein a 5% (w/v) GelMA hydrogel precursor solution was emulsified with microfluidic oil (paraffin oil containing 5 wt% Span 80) in a flow-focusing microfluidic device. Shear forces and hydrophobic interactions at the intersection point facilitated the formation of pre-gel droplets, which were subsequently cross-linked under UV irradiation. The microparticles were thoroughly rinsed with acetone and deionized water to eliminate residual oil and unreacted additives. Following fabrication, 1 mL of cell suspension (1 × 10^6^ cells/mL) was co-cultured with 2,000 hydrogel microparticles. After 3 days of incubation, the culture medium was replaced and maintained until the particles were collected on day seven for further experimental analysis. After pre-culturing for 24 h to allow cell adhesion, we replaced half of the culture medium (fresh DMEM supplemented with 10% FBS and 1% penicillin-streptomycin) every 12 h. All samples were collected after 7 days of cultivation for subsequent drug resistance experiments and studies.

### 2.4 Fabrication of the cell-encapsulated microgel

A novel droplet-based microfluidic 3D printing system was developed, comprising the following components: (a) syringe pump, (b) bioprinting machine arm, (c) operating system, (d) superhydrophobic surface, and (e) UV crosslinking equipment. Hydrogel precursors pre-mixed with Huh7 cells at a density of 1 × 10^6^ cells/mL, were loaded into syringes connected to the syringe pump. The precursor solutions were injected through the system, forming droplets via Plateau-Rayleigh instability. To stabilize the droplets, polydimethylsiloxane (PDMS) surfaces with micropillar structures were fabricated, mimicking the superhydrophobic characteristics of lotus leaves. This innovative setup enabled precise and efficient generation of cell-laden microdroplets for advanced 3D bioprinting applications. Subsequently, the morphology of microgels was observed under light and scanning electron microscopy (SEM).

### 2.5 Assessment of cytocompatibility

Live/Dead staining, CCK-8 assay, and HE staining were conducted to evaluate the cytocompatibility of the HCC-laden microparticles and microgels. Huh7 HCC-laden microparticles and microgels were prepared according to established protocols. Following 3 days of *in vitro* cultivation, the samples were incubated in the dark with 1 mL of Live/Dead staining solution for 30 min and subsequently observed under a laser scanning confocal microscope (Leica, Germany) to assess cell viability. Cell proliferation was evaluated at multiple time points over 7 days using the CCK-8 assay, following the manufacturer’s guidelines, with results expressed as mean optical density (OD) values. For histological analysis, the *in vitro* cultured cell-laden microparticles and microgels were embedded in paraffin, sectioned into slices approximately 5 μm thick, and stained with HE according to the manufacturer’s instructions. Stained sections were observed and imaged using an optical microscope (Olympus, Japan), enabling detailed examination of cell morphology and distribution.

### 2.6 Evaluation of chemoresistance

To investigate the impact of DHCM on chemoresistance in HCCs, we first assessed cell proliferation using a DNA quantification kit and evaluated histological changes in Huh7-encapsulated microgels cultured *in vitro* for 1 week. Gradient concentrations of 5-fluorouracil (5FU) were applied to Huh7 HCCs to determine the half-maximal inhibitory concentration (IC50), which was calculated using a DNA quantification assay. Specifically, the IC50 was determined by constructing a full dose-response curve. Cells were exposed to a range of 5FU concentrations (0–100 µM) and cell viability was assessed at 48 h using the DNA quantification assay. The dose-response curve was generated and the IC50 value was calculated through nonlinear regression analysis. This IC50 value was subsequently used in further *in vitro* chemoresistance assays. Huh7 HCCs were cultured in DHCM/GelMA and GelMA microgels and treated with 5FU for 7 days. To assess apoptosis rates, flow cytometry was performed using Annexin V-FITC and propidium iodide (PI) staining. This approach allowed for the differentiation of early apoptotic (Annexin V+/PI−), late apoptotic (Annexin V+/PI+), and necrotic (Annexin V−/PI+) cell populations. The analysis facilitated the evaluation of changes in chemoresistance to 5FU under different microenvironmental conditions. These experiments provided insights into the role of DHCM in modulating chemotherapeutic resistance at the *in vitro* level.

In animal chemotherapy studies, nude mice were randomly allocated to treatment or control groups using a computer-generated randomization table. Based on a power analysis (α = 0.05, power = 0.8), a sample size of n = 8 per group was determined to ensure statistical significance while adhering to ethical guidelines for animal use. Subsequently, Huh7 HCC-encapsulated DHCM/GelMA and GelMA microgels were subcutaneously injected into the axillary region of the nude mice. After 2 weeks of tumor establishment, mice with visible tumors were selected for a 5FU chemotherapy study. Intraperitoneal (i.p.) administration of 5FU was performed at a therapeutic dose of 100 mg/kg. During the procedure, mice were restrained in a supine position to facilitate accurate injection. Using a 27G needle, the drug was administered into the peritoneal cavity at a shallow angle, avoiding the midline to reduce the risk of puncturing internal organs. The injection volume was limited to 100 μL per dose to prevent peritoneal over-distension. Mice were monitored post-injection for adverse reactions such as discomfort, bloating, lethargy, or weight loss, which could indicate improper administration or drug-related toxicity. Following two cycles of 5FU administration, given every 3 days, tumors were harvested, sectioned, and analyzed for diameter measurements using a caliper and histological evaluation using HE staining.

### 2.7 Transmission electron microscopy (TEM)

The Huh7 HCCs were digested from the microgels and fixed in 2.5% glutaraldehyde overnight, followed by fixation in 2% osmium tetroxide for 1 h. Subsequently, the samples were stained with 2% uranyl acetate for 1 h. Dehydration was performed using an ascending series of acetone prior to embedding in araldite. Semi-thin sections were prepared and stained with toluidine blue to locate cells before observation under a transmission electron microscope (Hitachi, Tokyo, Japan).

### 2.8 Immunofluorescence staining

Following rehydration, tissue sections were blocked with goat serum and treated with 0.8% hyaluronidase for 20 min at 37°C. The sections were then incubated overnight at 4°C with primary antibodies. After washing, they were incubated with fluorescent dye-conjugated secondary antibodies for 2 h at room temperature. Finally, the sections were counterstained with DAPI and imaged using a laser scanning confocal microscope (Leica, Germany).

### 2.9 Statistical analysis

All experiments were performed in triplicate or more. Results are expressed as the mean ± standard deviation (SD). Statistical significance among groups was assessed using paired or unpaired t-test, with a threshold of P < 0.05 considered statistically significant.

## 3 Results

### 3.1 Preparation and characterization of photo-crosslinked DHCM hydrogel microgels and microparticles

**FIGURE 1:**
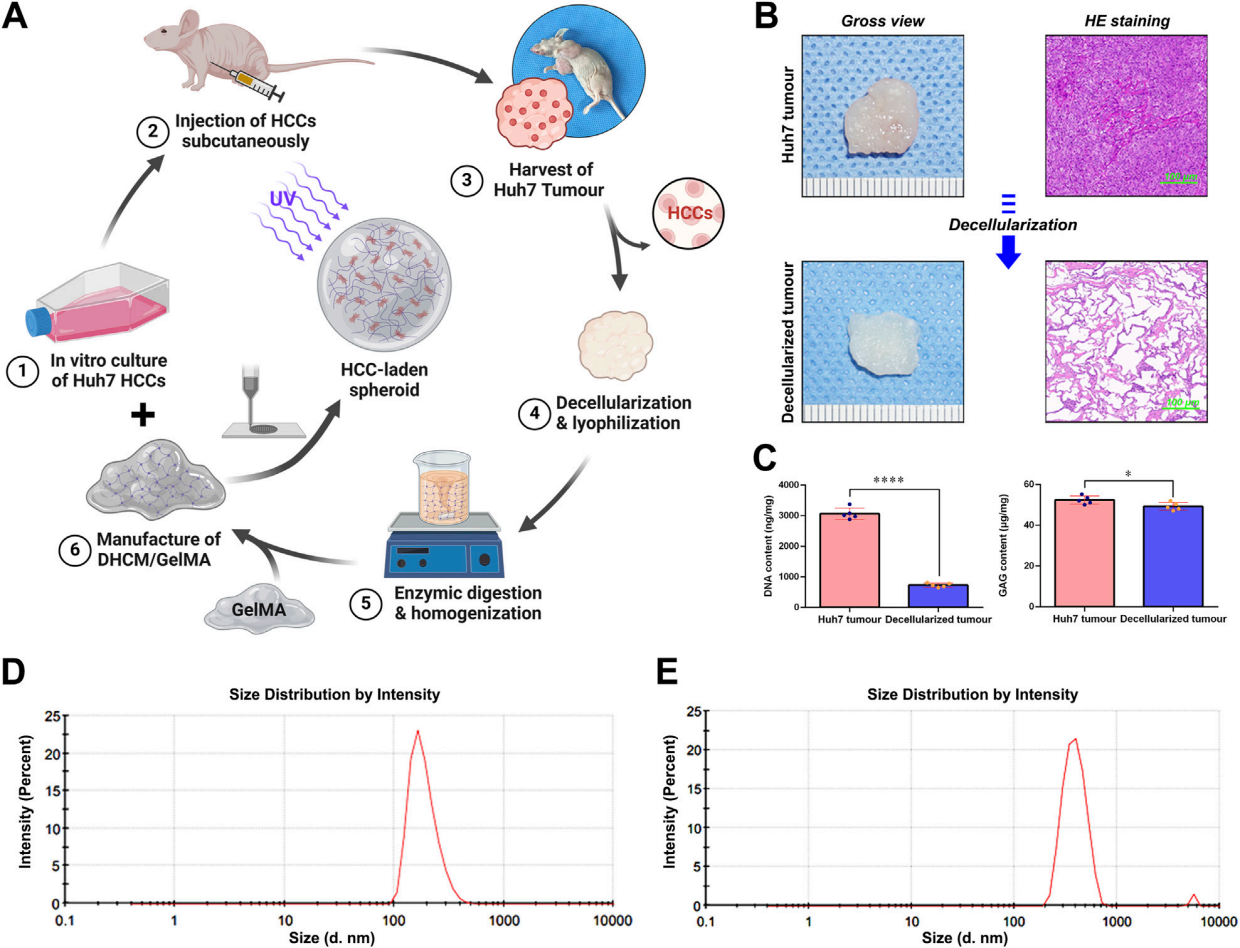
Schematic representation of the synthesis workflow for HCC-laden microgels and the subsequent characterization of DHCM. **(A)** Schematic workflow for development of HCC-laden microgels. **(B)** Gross view and HE staining of the freshly harvested hepatocellular carcinoma and DHCM tissues. **(C)** Statistical analysis of the DNA and GAG contents of the freshly harvested hepatocellular carcinoma and DHCM tissues. **(D)** Analysis of particle size distribution of GelMA hydrogel precursors. **(E)** Analysis of particle size distribution of DHCM/GelMA hydrogel precursors. ****p < 0.0001, *p < 0.05.

According to the workflow illustrated in Figure 1A, Huh7 HCCs were expanded and cultured to develop subcutaneous tumors in nude mice. After 2 weeks, tumors were excised and subjected to chemical decellularization to generate DHCM. This DHCM was enzymatically processed into a gel precursor, which was then combined with GelMA photo-crosslinked hydrogel to produce DHCM/GelMA hydrogels enriched with ECM components. In brief, hydrogels were prepared by incorporating varying concentrations of DHCM (0%, 3%, 5%, and 8% w/v) into a 5% w/v GelMA hydrogel, with 0.25% w/v LAP serving as the crosslinking agent, following established protocols for hydrogel microsphere preparation (Hua et al., 2022). However, when the DHCM concentration reached 8% w/v, a significant reduction in crosslinking efficiency was observed (Supplementary Data). As a result, a formulation of 5% w/v DHCM, 5% w/v GelMA, and 0.25% w/v LAP was chosen for optimal hydrogel preparation, ensuring both effective crosslinking and maximal incorporation of DHCM. These hydrogels were used to fabricate microgels and microparticles. HE staining (Figure 1B) confirmed effective removal of cellular material, retaining only the ECM. The results of quantification of the DNA content in the fresh and decellularized hepatocellular carcinoma tumor samples revealed that this strategy achieved satisfactory decellularization outcomes (Figure 1C). The contents of glycosaminoglycans (GAG), key components of tumor matrix, were assessed using quantitative ELISA. The results indicated that the GAG contents were slightly lower in the decellularized hepatocellular carcinoma tumor samples than in the fresh tumor samples (Figure 1C). Analysis of particle size distribution showed an average particle size of approximately 300 nm for GelMA hydrogel precursors (Figure 1D), which increased to around 700 nm when mixed with DHCM hydrogel precursors (Figure 1E).

**FIGURE 2:**
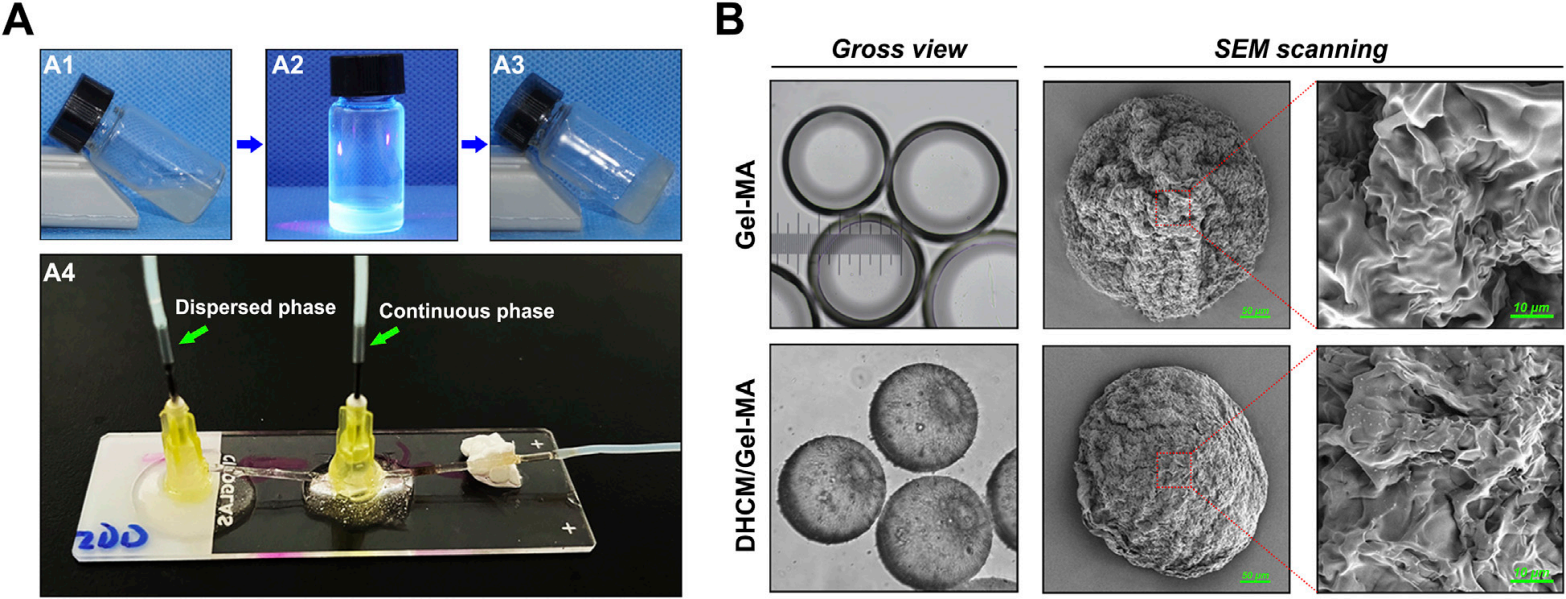
Fabrication and characterization of microparticles synthesized via droplet microfluidics. **(A)** Gelationprocess of DHCM/GelMA hydrogel under UV irradiation and image of the microfluidic device utilized in the synthesis. **(B)** Gross morphology and SEM imaging of microparticles fabricated through droplet microfluidics.

Microparticles were fabricated using droplet microfluidics (Figure 2A), where oil-phase cutting generated hydrogel precursor droplets that self-assembled into spherical shapes under surface tension and were subsequently crosslinked with UV light. Microscopy and SEM imaging (Figure 2B) demonstrated the spherical morphology, reduced transparency, and ECM richness of the DHCM/GelMA microparticles.

**FIGURE 3:**
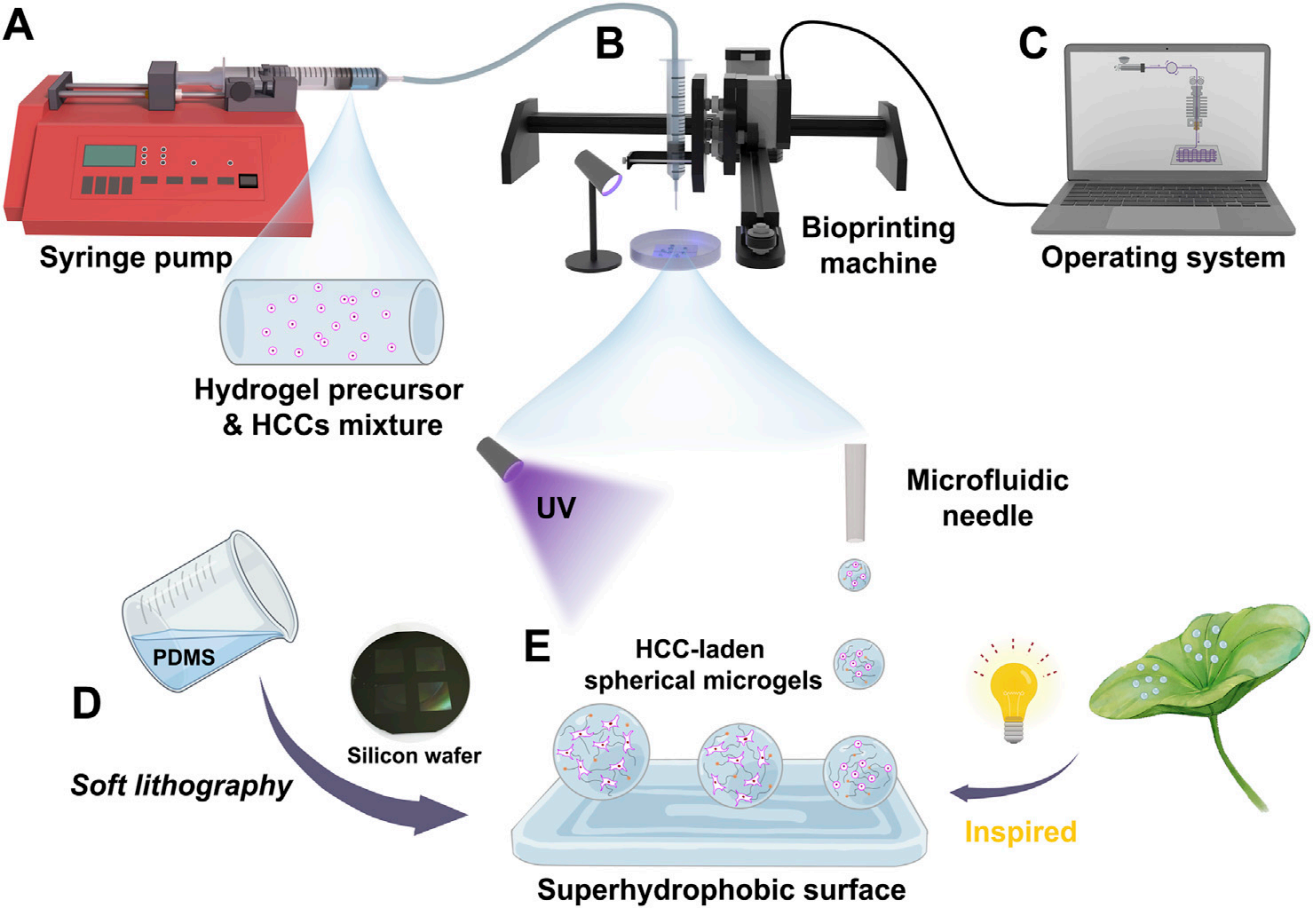
Schematic representation of the fabrication process for HCC-encapsulated microgels using a customized droplet microfluidic 3D printing system. **(A)** Injection of GelMA or DHCM/GelMA precursors pre-mixed with Huh7 cells via syringe pumps. **(B)** Droplet formation through the bioprinting system. **(C)** Control of the bioprinting system via an integrated operating interface. **(D)** Fabrication of a PDMS-based superhydrophobic surface using a soft lithography approach. **(E)** Formation of HCC-encapsulated microgels under UV irradiation.

**FIGURE 4:**
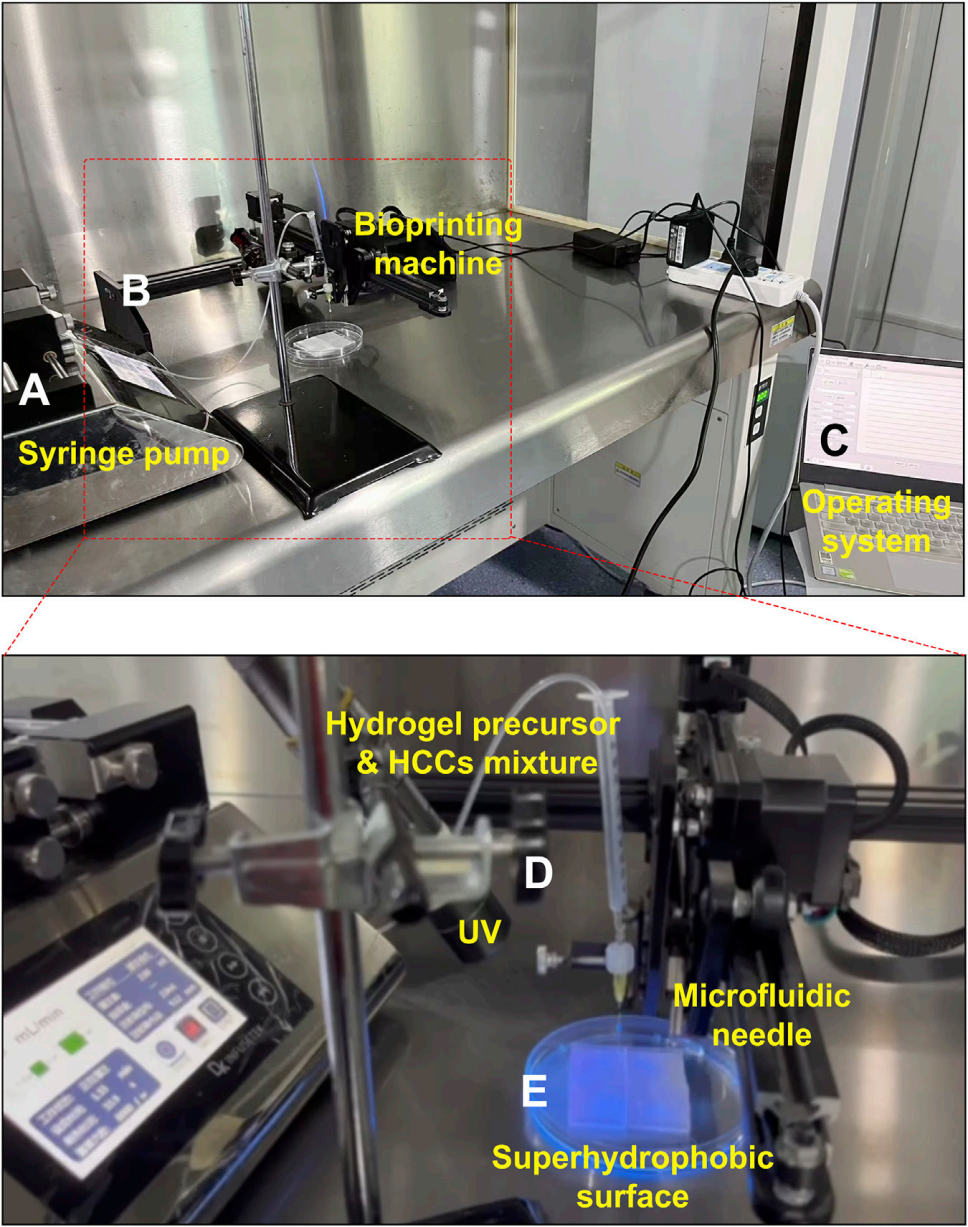
Key components of the customized droplet microfluidic 3D printing system. **(A)** Syringe pumps. **(B)** Bioprinting system. **(C)** Operating interface. **(D)** UV irradiator. **(E)** Superhydrophobic surface.

Further, HCCs-encapsulated microgels were fabricated using a customized droplet microfluidic 3D printing system (Figures 3, 4). GelMA or DHCM/GelMA precursors, pre-mixed with Huh7 cells, were delivered into the 3D printing system via syringe pumps (Figures 3A, B). Droplet formation was achieved through Plateau-Rayleigh instability, ensuring uniformity in size and structure (Figure 3C). The bioprinting arms were precisely controlled by an integrated operating system to ensure consistency and accuracy during the process (Figure 4C). To stabilize the droplets, superhydrophobic PDMS surfaces with micropillar structures were engineered, drawing inspiration from the lotus leaf’s natural superhydrophobic properties (Figures 3D, E). This innovative system effectively combined biological and engineering principles to produce stable, high-quality microgels for subsequent experimentation.

**FIGURE 5:**
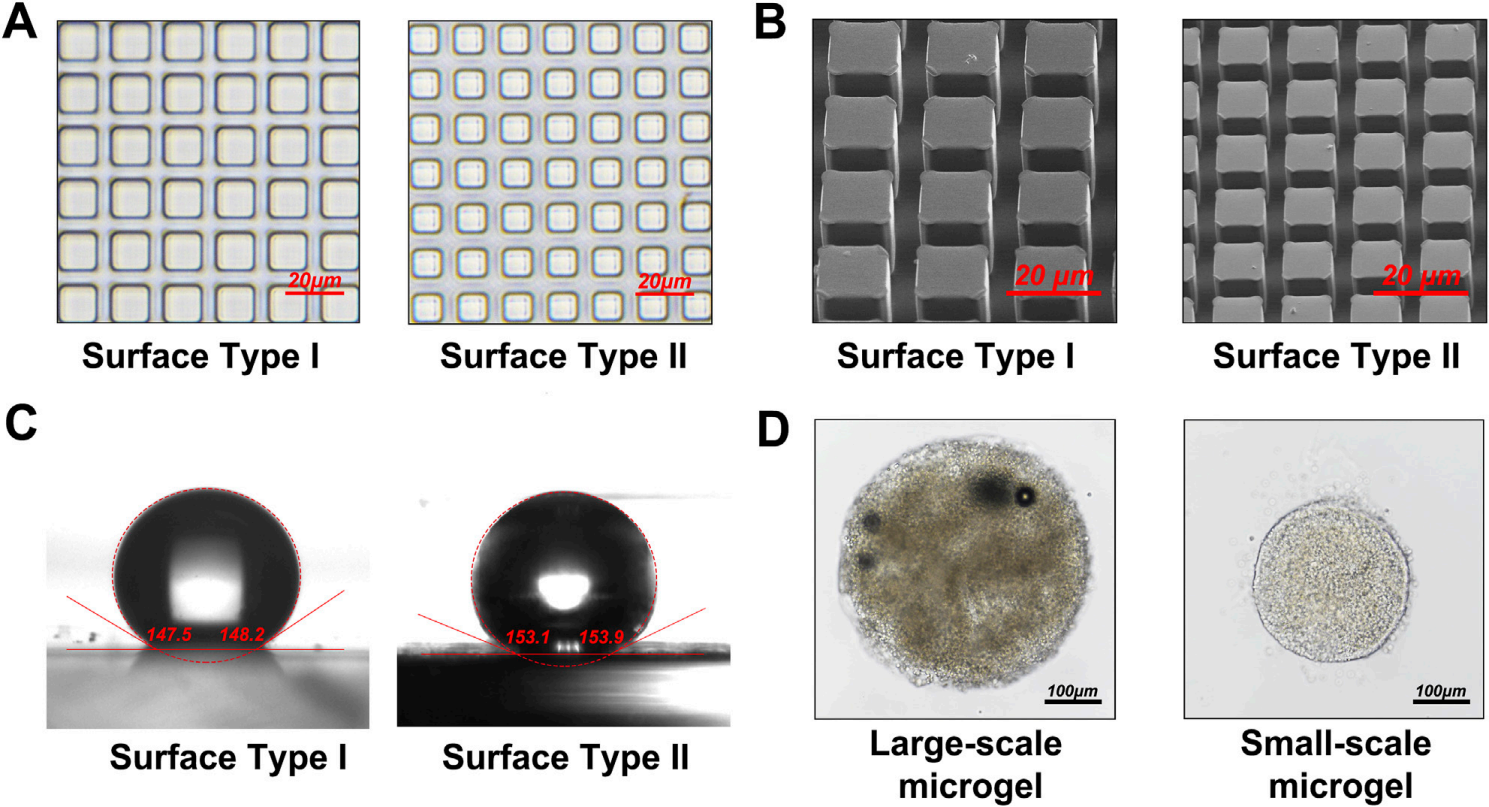
Examination of the superhydrophobic surface and microgel morphology. **(A)** Optical microscopy images of the PDMS superhydrophobic surface featuring micropillars with diameters of 5 μm and 15 µm. **(B)** SEM micrographs of the PDMS superhydrophobic surface with 5 μm and 15 µm micropillars. **(C)** Measurement and analysis of contact angles on the PDMS superhydrophobic surface with 5 μm and 15 µm micropillars. **(D)** Generation of microgels with diameters ranging from 200 μm to 500 µm by adjusting microfluidic flow parameters.

Microgels fabricated on surfaces with 5 μm and 15 µm micropillars exhibited distinct morphologies under light and electron microscopy (Figures 5A, B). Surfaces with 5 µm pillars achieved contact angles greater than 150°, forming ultra-hydrophobic interfaces (Figure 5C). By modulating microfluidic flow conditions, microgels with diameters ranging from 200 μm to 500 µm were produced (Figure 5D), rendering them suitable for various tissue engineering and organoid applications.

### 3.2 Construction of engineered hepatocellular carcinoma microtissues

**FIGURE 6:**
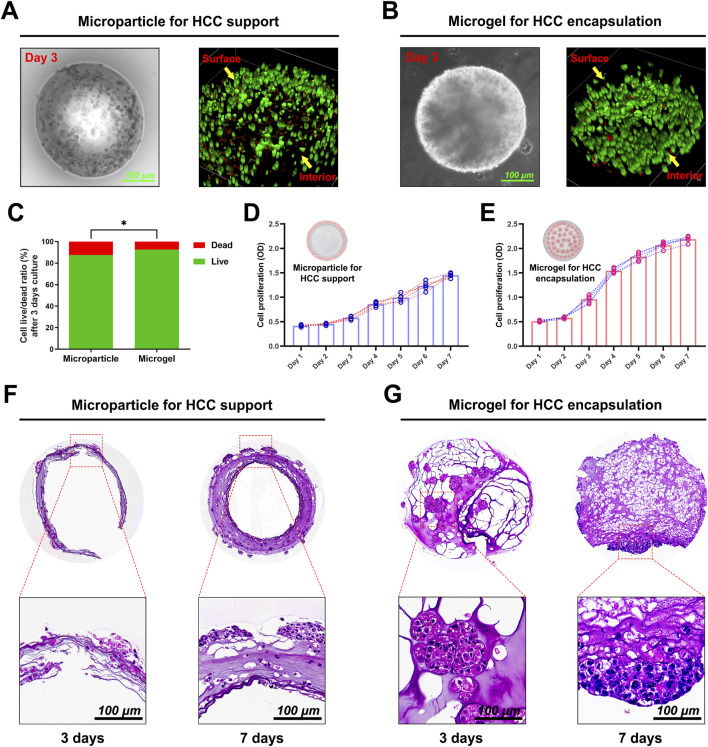
Preparation and cultivation of HCC-laden microparticles and microgels. **(A)** Live/dead staining of HCC-laden microparticles after 3 days of *in vitro* culture. **(B)** Live/dead staining of HCC-encapsulated microgels after 3 days of *in vitro* culture. **(C)** Quantitative analysis of live/dead staining results for both microparticles and microgels. **(D)** Proliferation curve of HCCs embedded in microparticles over a 1-week *in vitro* culture period. **(E)** Proliferation curve of HCCs encapsulated in microgels over a 1-week *in vitro* culture period. **(F)** HE staining of HCC-laden microparticles after 3 and 7 days of culture. **(G)** HE staining of HCC-encapsulated microgels after 3 and 7 days of culture. *p < 0.05.

Huh7 HCCs were encapsulated in GelMA or DHCM/GelMA hydrogels at a density of 1 × 10^6^ cells/mL to fabricate microgels and microparticles. Fluorescent imaging after 3 days of *in vitro* culture revealed distinct cell distribution patterns: cells in microparticles were predominantly located on the surface (Figure 6A), whereas microgels enabled a uniform distribution of cells throughout both the interior and surface regions (Figure 6B). Furthermore, microgels exhibited significantly higher cell viability compared to microparticles, highlighting their superior biocompatibility and suitability for cell encapsulation applications (Figure 6C).

Proliferation assays indicated sustained cell growth over a week in both systems, with microgels exhibiting a steeper proliferation curve and enhanced metabolic activity (Figures 6D, E). Histological analysis at days 3 and 7 (Figures 6F, G) showed minimal cellular infiltration within microparticles, whereas microgels supported extensive internal cellular proliferation and uniform tissue organization. These results underscore the superior capability of microgels for creating engineered hepatocellular carcinoma microtissues, positioning them as a promising platform for drug screening and organoid culture applications.

### 3.3 DHCM enhances chemoresistance in Huh7 HCCs

**FIGURE 7:**
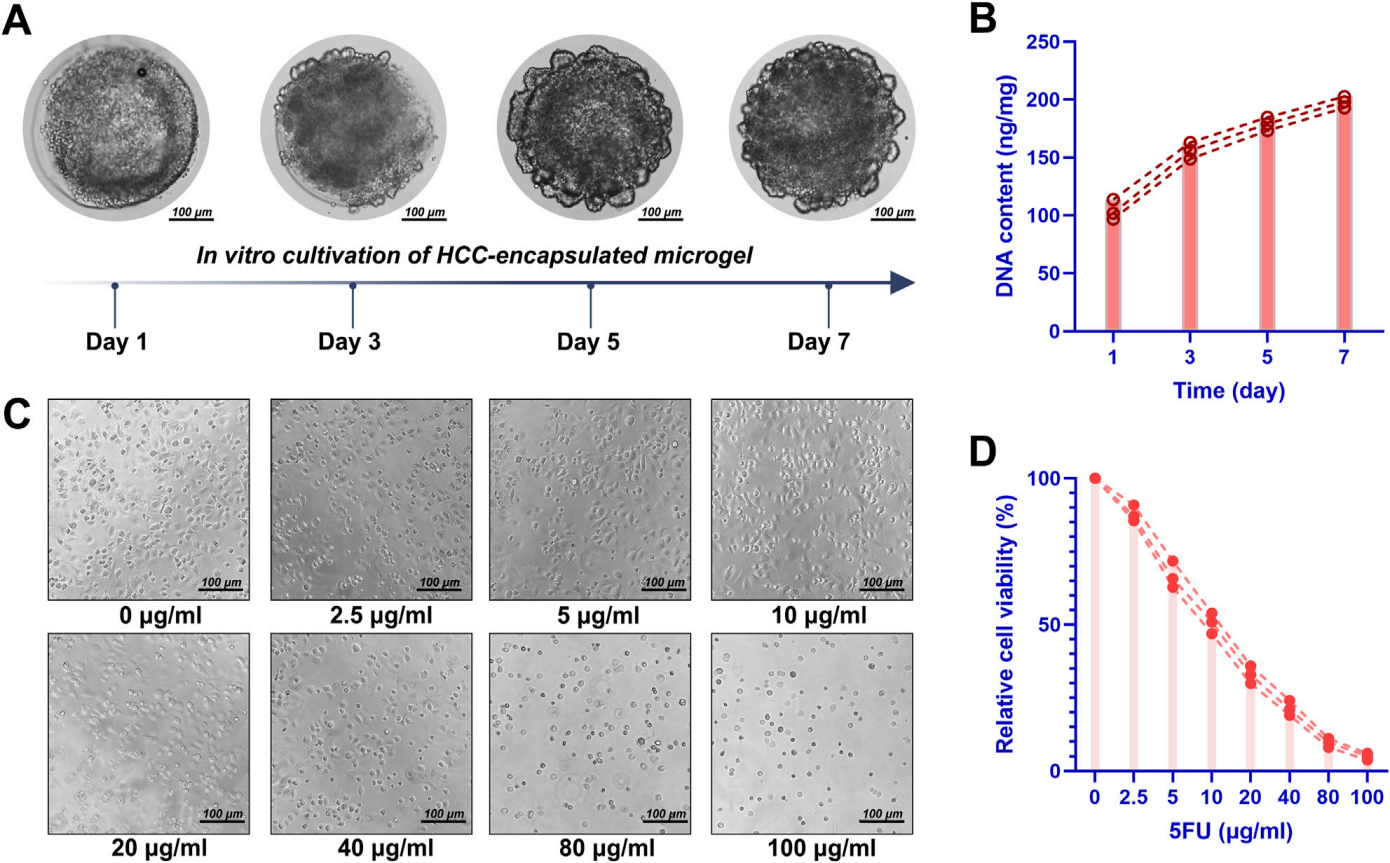
Cultivation of HCC-laden DHCM/GelMA microgels. **(A)** Optical observation of HCC-laden DHCM/GelMA microgels following 7 days of *in vitro* culture. **(B)** DNA quantification of HCC-laden DHCM/GelMA microgels after 7 days of *in vitro* culture. **(C)** Observations of HCCs cultured in DHCM/GelMA microgels under graded concentrations of 5-fluorouracil (5FU), with quantitative analysis of live/dead staining results. **(D)** Viability analysis and proliferation curve of HCCs embedded in DHCM/GelMA microgels during 1 week of *in vitro* culture under 5FU treatment.

**FIGURE 8:**
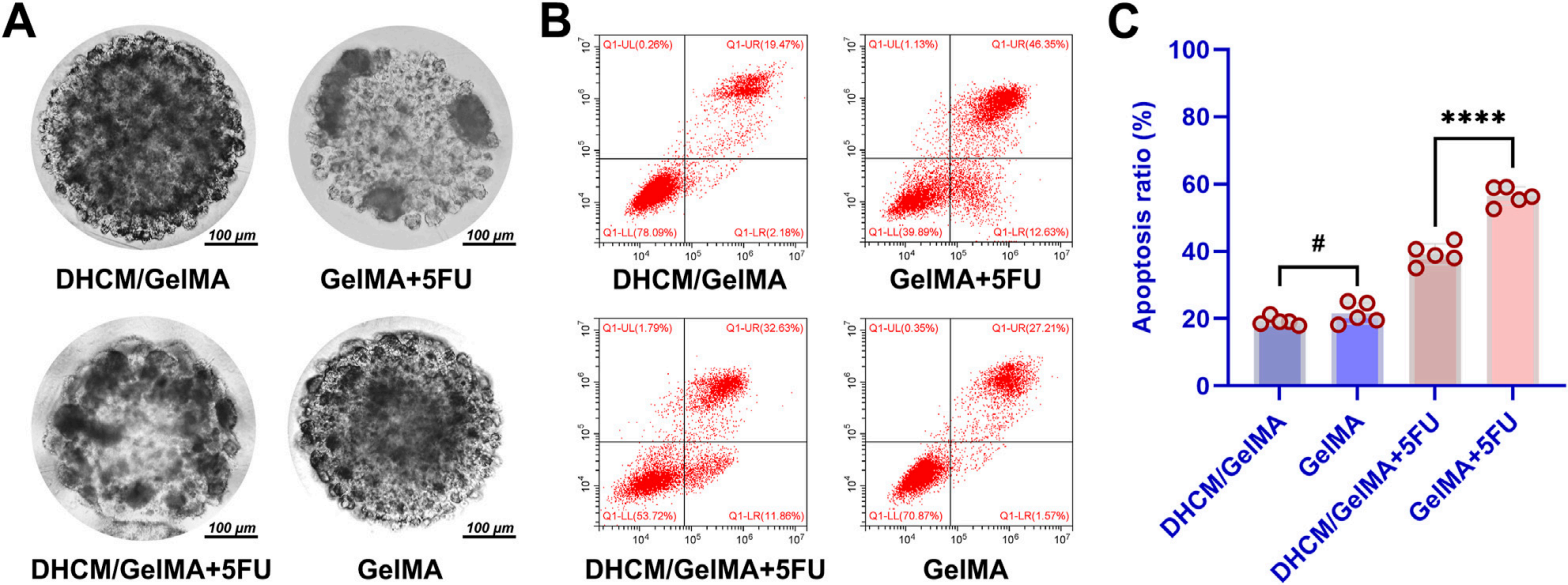
*In vitro* evaluation of chemoresistance variations in HCC-laden microgels. **(A)** Microscopic comparison of cell numbers and distribution within GelMA microgels and DHCM/GelMA microgels. **(B)** Flow cytometry analysis of apoptosis rates in GelMA microgels versus DHCM/GelMA microgels. **(C)** Statistical analysis of apoptosis rates between GelMA and DHCM/GelMA microgels, with significance levels indicated. ****p < 0.0001, ^#^p > 0.05.

Dynamic observations demonstrated that DHCM/GelMA hydrogels were largely replaced by tumor-derived ECM within 5–7 days of culture (Figures 7A, B), exhibiting tumor formation rates close to GelMA hydrogels. To assess chemoresistance, Huh7 cells encapsulated in GelMA or DHCM/GelMA hydrogels were exposed to 10 μg/mL 5FU. Microscopic analysis revealed a more pronounced reduction in cell numbers within GelMA microgels compared to DHCM/GelMA microgels (Figure 8A). Flow cytometry further confirmed significantly higher apoptosis rates in GelMA microgels relative to DHCM/GelMA counterparts (Figures 8B, C).

**FIGURE 9:**
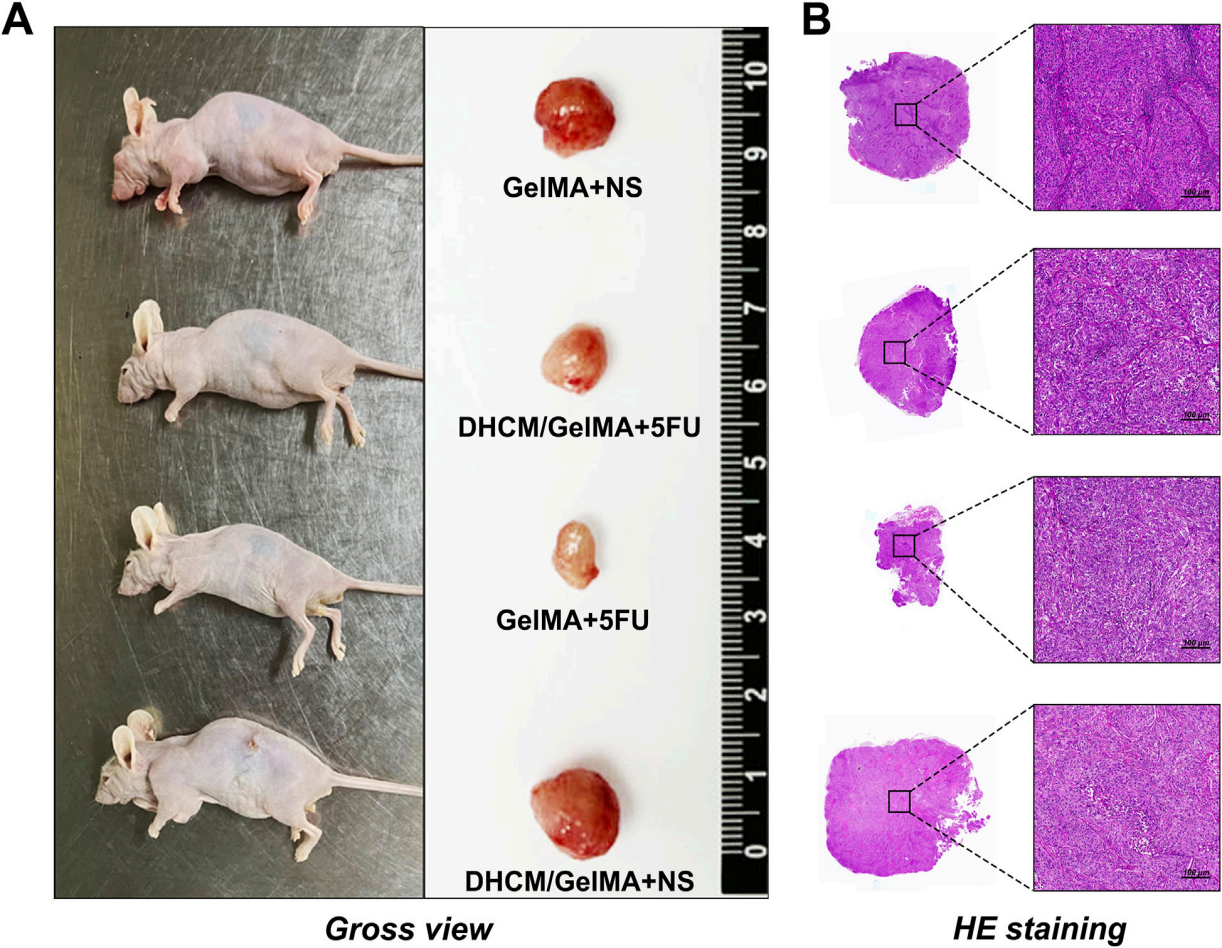
*In vivo* evaluation of chemoresistance in HCC-laden microgels. **(A)** Macroscopic images of tumors derived from DHCM/GelMA and GelMA microgels after subcutaneous implantation in nude mice followed by 5FU treatment. **(B)** HE staining of tumors derived from DHCM/GelMA and GelMA microgels post-5FU treatment in subcutaneously implanted nude mice.

*In vivo* studies corroborated these findings. Following subcutaneous implantation in nude mice, tumors derived from DHCM/GelMA microgels displayed larger volumes after 5FU treatment than those derived from GelMA microgels (Figures 9A, B). These results demonstrate that the incorporation of DHCM significantly enhances the chemoresistance of Huh7 HCCs.

### 3.4 Mechanism of DHCM-induced chemoresistance: AQP3-mediated autophagy

**FIGURE 10:**
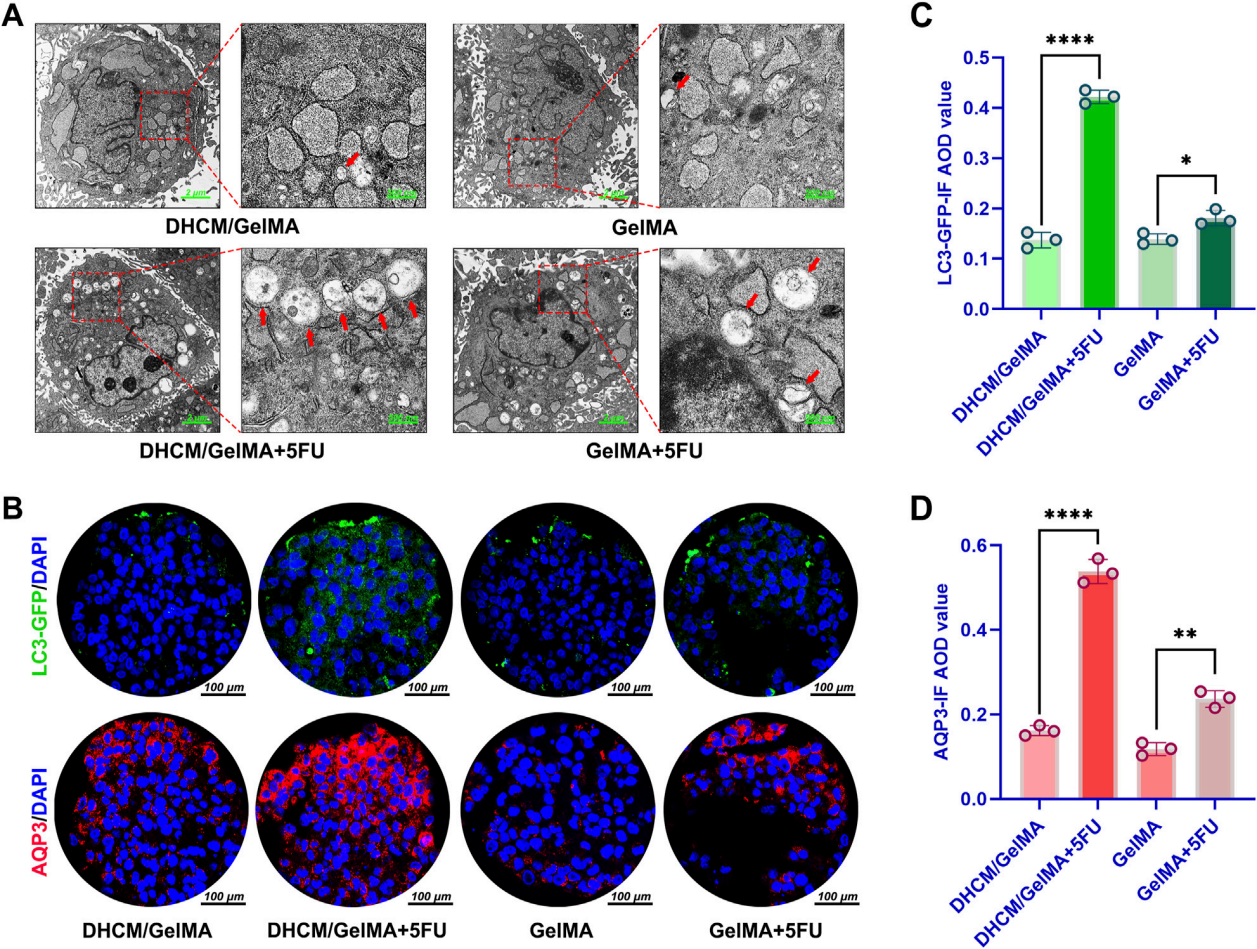
Evaluation of autophagy activity induced by DHCM. **(A)** Transmission electron microscopy (TEM) images of Huh7 cells encapsulated in GelMA or DHCM/GelMA hydrogels following 5FU exposure. **(B)** IF staining of LC3 (green) and AQP3 (red) in Huh7 cells encapsulated in GelMA or DHCM/GelMA hydrogels after 5FU treatment. **(C)** Quantitative analysis of LC3 IF average optical density (AOD) values in Huh7 cells encapsulated in GelMA or DHCM/GelMA hydrogels following 5FU exposure. **(D)** Quantitative analysis of AQP3 IF AOD values in Huh7 cells encapsulated in GelMA or DHCM/GelMA hydrogels following 5FU exposure. *p < 0.05, **p < 0.01, and ****p < 0.0001.

Previous studies have established a strong association between aquaporin 3 (AQP3) and the activation of autophagy, as well as chemoresistance, mediated through the modulation of the mTOR signaling pathway (Liang et al., 2024). Additionally, the pathway enrichment data provided in the Supplementary Material also offered some clues suggesting that DHCM upregulates AQP3 and further activates mTOR pathway-associated autophagy. TEM analysis in the current study revealed a marked increase in autophagosome formation in HCCs encapsulated within DHCM/GelMA microgels following treatment with 5FU (Figure 10A). This observation suggests enhanced autophagic activity under chemotherapeutic stress. Further validation using IF staining confirmed an increase in AQP3 and LC3-GFP expression in DHCM/GelMA microgels treated with 5FU (Figures 10B–D). Additionally, elevated LC3 expression indicated enhanced autophagic activity in these hydrogels under chemotherapeutic stress.

To further validate these findings, IF staining was employed to evaluate the expression levels of AQP3 and the autophagy marker LC3-GFP. The results confirmed a significant upregulation of both AQP3 and LC3-GFP in DHCM/GelMA microgels subjected to 5FU treatment (Figures 10B–D). Specifically, the increased expression of LC3, a hallmark of autophagosome formation, provided additional evidence of heightened autophagic activity within the DHCM-based hydrogels under chemotherapy-induced stress conditions.

These findings indicate that the incorporation of DHCM into GelMA hydrogels significantly enhances chemoresistance in HCCs. The observed mechanism involves the upregulation of AQP3 expression, which activates autophagy pathways that mitigate the cytotoxic effects of chemotherapeutic agents such as 5FU. Autophagy, in this context, functions as a protective response, promoting cellular survival by clearing toxic intracellular components and reducing apoptosis.

## 4 Discussion

The mechanisms underlying drug resistance in hepatocellular carcinoma and the development of personalized therapeutic strategies are increasingly pivotal in cancer research. The FDA’s recent endorsement of organoids as platforms for drug screening, encompassing drug sensitivity and resistance testing, has significantly advanced the construction and cultivation of liver cancer microtissues and organoids (Vernetti et al., 2016; Nuciforo and Heim, 2021).

Traditionally, culturing HCCs on microparticles fabricated using conventional microfluidic techniques has encountered numerous challenges, such as inadequate biocompatibility, mismatches between material degradation and cellular proliferation rates, and limited cell migration into the carrier interior (Murphy and Atala, 2014; Sun et al., 2020). Our research highlighted similar limitations, revealing that HCCs cultured on GelMA hydrogel microparticles displayed restricted or absent migration into the hydrogel matrix. This led to the formation of tumor-like aggregates on the carrier surface, impeding the accurate replication of *in vivo* liver cancer tissue characteristics and compromising the reliability of drug resistance studies.

In recent years, advances in 3D bioprinting have allowed researchers to encapsulate cells within gel-like materials for direct fabrication of cell-laden microgels, yielding promising results for microtissue and organoid cultivation (Sun et al., 2020; Zhuang et al., 2023). However, the high precision required for printing microstructures, particularly regarding mechanical arm and nozzle accuracy, remains a significant barrier to the widespread clinical application of this technology (Liu et al., 2023).

Drawing inspiration from the stable microdroplets observed on lotus leaves, we employed droplet microfluidics based on Plateau-Rayleigh instability to establish a novel, tunable method for fabricating cell-laden microgels. This approach integrates droplet microfluidics, 3D printing, and superhydrophobic surface techniques to enable the efficient, cost-effective, and scalable production of cell-laden microgels. Plateau-Rayleigh instability describes how surface tension causes a liquid column to fragment into droplets when the wavelength of surface fluctuations surpasses the column circumference.

Inspired by the stable microdroplets observed on lotus leaves, we employed droplet microfluidics based on Plateau-Rayleigh instability to fabricate cell-laden microgels. This approach integrates droplet microfluidics, 3D printing, and superhydrophobic surface techniques, enabling efficient, cost-effective, and scalable production of cell-laden microgels. Plateau-Rayleigh instability describes the process by which surface tension causes a liquid column to fragment into droplets when the wavelength of surface fluctuations exceeds the column circumference (Xue et al., 2016). In theory, surfaces with finer textures are expected to exhibit superior hydrophobicity (Le Donne et al., 2023). However, our study is constrained by the limitations of current soft lithography technology, which offers a maximum precision of 5 µm. As a result, we focused on 5 µm micropillars, representing the highest resolution achievable with our existing platform. Additionally, while specialized coatings could potentially enhance hydrophobicity, the biocompatibility of such materials requires thorough evaluation, which is beyond the scope of this study.

Given these limitations and the need for biocompatible materials, we selected 5 µm pillar-structured PDMS for microgel fabrication. We recognize that future investigations could explore a broader range of feature dimensions and coating materials to further optimize the hydrophobic properties and functional performance of the microgels. To maintain the spherical morphology of droplets post-crosslinking, we developed a superhydrophobic interface inspired by the lotus leaf effect, utilizing its self-cleaning and water-repellent properties (Sharma et al., 2021). By dispensing photocrosslinkable hydrogel precursor droplets onto this interface in a controlled arrangement, we used hydrophobic interactions to form uniformly spherical droplets, which were subsequently crosslinked under UV light to create stable cell-encapsulated hydrogel systems. This method offers a versatile 3D printing-based approach for producing microgel systems applicable to *in vitro* tissue engineering, including microtissues and organoids, as well as drug sensitivity and resistance studies.

Using this platform, hepatocellular carcinoma microtissues were constructed and cultured based on HCC-encapsulated DHCM/GelMA and GelMA microgels, enabling investigations into chemoresistance. The results revealed that integrating hepatocellular carcinoma ECM components into GelMA hydrogels significantly enhanced HCC chemoresistance. ECM-driven mechanisms play a critical role in hepatocellular carcinoma chemoresistance through a variety of interconnected pathways (Lu et al., 2012; Nallanthighal et al., 2019; Girigoswami et al., 2021). The dense structure of the ECM acts as a physical barrier, limiting drug penetration and lowering local drug concentrations (Najafi et al., 2019; Ferrara et al., 2021; Wei et al., 2021). Additionally, ECM-integrin interactions activate anti-apoptotic signaling pathways such as PI3K/Akt and MAPK, inducing cell adhesion-mediated drug resistance (CAM-DR) (Jakubzig et al., 2018; Sidhu et al., 2021). ECM remodeling by tumor-associated fibroblasts (CAFs), which secrete resistance-promoting cytokines like TGF-β and VEGF, further modifies the tumor microenvironment (Liao et al., 2019; Guo and Xu, 2024). Specific ECM components modulate Wnt/β-catenin and Notch pathways, inducing stem cell-like properties in HCCs and increasing drug tolerance (Ding et al., 2015; Guo and Xu, 2024). Abnormal ECM-regulated vasculature decreases drug delivery efficiency, while altered enzyme activity, such as CYP450, accelerates drug degradation (Zhan et al., 2022). Moreover, ECM-driven hypoxia and oxidative stress activate autophagy pathways that protect cancer cells from drug cytotoxicity (Liu et al., 2018; Li et al., 2023). ECM-enhanced secretion of extracellular vesicles carrying resistance-associated molecules, such as miRNAs and proteins, facilitates paracrine signaling, promoting the expansion of resistant cell populations (Duan et al., 2024).

In our study, DHCM was processed into nanoscale ECM particles and mixed with photocrosslinkable GelMA hydrogels. Although this processing disrupted the structural integrity of the ECM, it preserved its functional components. GelMA hydrogels, recognized for their biocompatibility and ability to mimic ECM properties, were combined with hepatocellular carcinoma ECM to further investigate its role in chemoresistance. Molecular analyses revealed that AQP3-mediated autophagy is a key mechanism driving this resistance. AQP3 plays a critical role in liver cancer chemoresistance by enhancing autophagy through multiple mechanisms. Specifically, AQP3-induced autophagy suppresses apoptosis, thereby promoting cell survival during chemotherapy. Moreover, autophagy mitigates the effects of chemotherapy by clearing toxic metabolites and neutralizing chemotherapeutic agents such as doxorubicin. Our findings demonstrated that ECM components significantly upregulate AQP3 expression in HCCs. This upregulation activates autophagy, thereby enhancing resistance to 5FU. The implications of this study are twofold. First, the data underscore the critical role of the ECM in modulating tumor cell behavior, particularly in fostering chemoresistance. Second, the DHCM-based biomimetic model represents a powerful tool for investigating the molecular mechanisms underlying chemoresistance and for developing targeted therapeutic strategies. By providing a platform to study the interplay between the tumor microenvironment and chemoresistance, this model offers promising avenues for the identification of novel interventions aimed at overcoming drug resistance in hepatocellular carcinoma. These results underscore the pivotal role of ECM in modulating chemoresistance and provide insights into potential therapeutic targets for overcoming drug resistance in liver cancer.

This research provides a foundational understanding of ECM-induced drug resistance mechanisms and offers theoretical insights for developing targeted therapies. However, further investigation is necessary to elucidate the precise regulation of AQP3-mediated autophagy by hepatocellular carcinoma ECM and its therapeutic implications for overcoming chemoresistance. Although this study presents several novel findings, some limitations remain. For instance, it is well-established that superhydrophobic interfaces are created by rough surfaces with regular or irregular microstructures, leading to high contact angles and roll-off angles for surface droplets (typically greater than 150°). In theory, more finely textured surfaces should provide enhanced hydrophobicity. However, our research is constrained by the limitations of the current soft lithography technology, which restricts precision to 5 µm. As a result, we focused on 5 µm micropillars, representing the highest achievable resolution with our existing platform. Furthermore, during the early stages, when cells had not yet secreted sufficient ECM to form hepatocellular carcinoma microtissues, tissue sections often detached during preparation. We also attempted quantitative analysis of ECM components, but due to the high ECM content in the DHCM/GelMA hydrogel, it was challenging to demonstrate statistically significant differences in the quantitative data as the newly formed ECM replaced the original ECM within the material. As a result, analyzing the dose-response relationship of ECM deposition using this method proved difficult. This limitation underscores the need for further investigation in future studies.

## 5 Conclusion

Altogether, the present study employed DHCM as a substrate, hybridizing it with GelMA hydrogel precursors to create a biomimetic scaffold enriched with hepatocellular carcinoma ECM components. By modulating the scaffold’s physical properties, the design achieved dual biomimicry, encompassing both physicochemical characteristics and functional composition. Utilizing a custom-built microfluidic 3D instrument, the study enabled the efficient production of cell-encapsulated microgels, which facilitated the *in vitro* construction of hepatocellular carcinoma microtissues for investigating drug resistance mechanisms. The results revealed that the decellularized ECM markedly enhanced Huh7 HCC chemoresistance, primarily through the upregulation of AQP3 expression and the subsequent activation of autophagy. This innovative approach provides a robust platform and identifies potential regulatory targets for advancing research into liver cancer drug resistance and the development of novel therapeutic interventions.

## Data Availability

The raw data supporting the conclusions of this article will be made available by the authors, without undue reservation.
