# Supplementary figures and images for: A novel approach for engineering DHCM/GelMA microgels: application in hepatocellular carcinoma cell encapsulation and chemoresistance research
Source: Front Bioeng Biotechnol. 2025 Mar 14;13:1564543. doi: 10.3389/fbioe.2025.1564543 (PMC11949893; doi:10.3389/fbioe.2025.1564543)

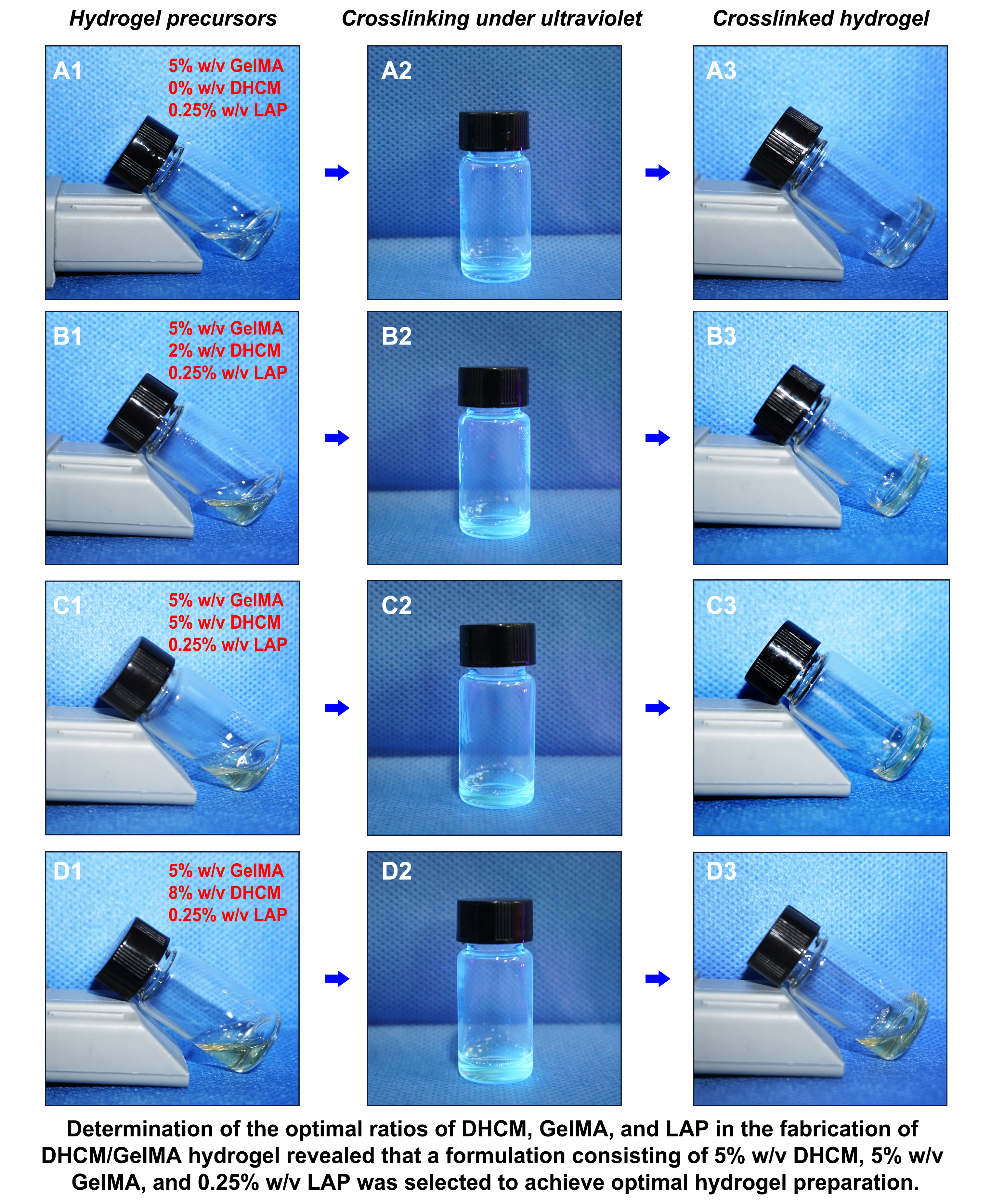

Supplement: Supplementary file 1 [file Image2.tif]

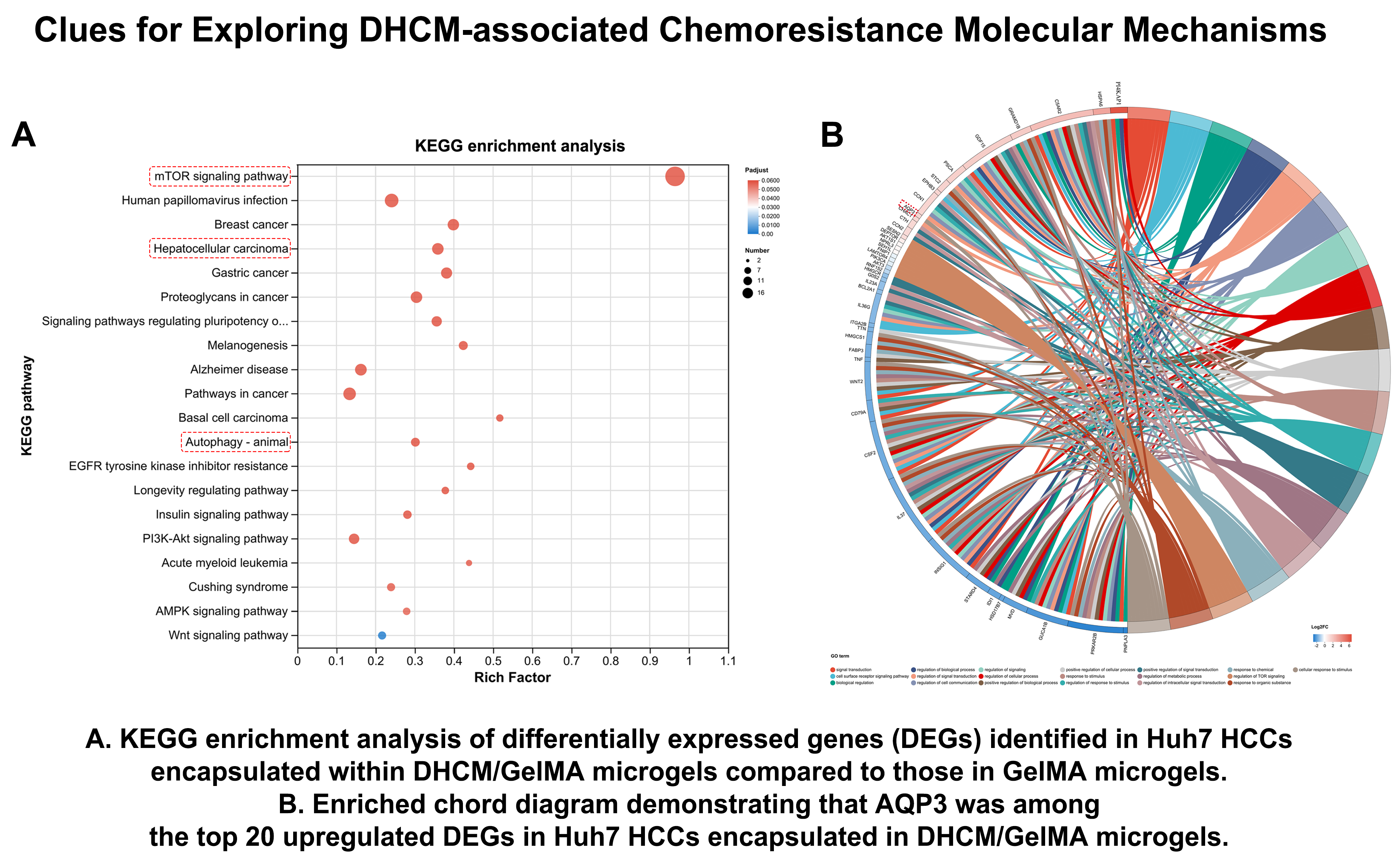

Supplement: Supplementary file 2 [file Image1.tif]
